# Cutaneous Protothecosis: Reported Human Cases in Vietnam and Diagnostic Challenges

**DOI:** 10.1155/crdm/9136305

**Published:** 2026-10-09

**Authors:** Thuy Thi Phan Nguyen, Ba Tong Tran, Linh Pham Thi Thuy, Hoang Vu Nguyen, Nhi Thi Uyen Pham

**Affiliations:** ^1^ Ho Chi Minh City Hospital of Dermato-Venereology Ho Chi Minh City Vietnam; ^2^ Faculty of Medicine University of Health Sciences, Viet Nam National University Ho Chi Minh City Vietnam

**Keywords:** cutaneous infections, histopathological examination, opportunistic infection, Prototheca species, protothecosis

## Abstract

Protothecosis, a rare opportunistic infection caused by *Prototheca* species, achlorophyllous microalgae, is increasingly recognized but remains underdiagnosed due to its rarity and nonspecific clinical presentation. We report two cases of human protothecosis in Vietnam, occurring in a 63‐year‐old and 81‐year‐old female presenting with chronic, painful, ulcerative lesions on upper extremities. Despite initial misdiagnosis and empirical antifungal and antibacterial treatments, histopathological examination revealed characteristic *Prototheca* sporangia, leading to the definitive diagnosis. These cases underscore the importance of considering protothecosis in atypical cutaneous infections, particularly when patients fail to respond to empirical antibacterial or antifungal therapies, and highlight diagnostic and treatment challenges.

## 1. Introduction

Protothecosis is an uncommon infection caused by the *Prototheca* species, which are unicellular, achlorophyllous, saprophytic microalgae. Members of the genus *Prototheca (Trebouxiophyceae* and *Chlorophyta)* are unicellular, nonphotosynthetic, and saprophytic microalgae that are phylogenetically closely related to the photosynthetic green algae of the genera *Chlorella* and *Auxenochlorella* [1, 2]. Having lost their photosynthetic activity during the evolutionary transition from autotrophy to heterotrophy, these organisms lack chlorophyll but retain degenerate, colorless plastids [1]. Structurally, they are characterized by a highly durable cell wall containing sporopollenin, which provides exceptional resistance to environmental stressors, desiccation, and standard disinfection practices [2]. Historically contentious, the taxonomy of *Prototheca* has been significantly revised in recent years through molecular and phylogenetic analyses, particularly using the mitochondrial apocytochrome B (cytb) gene as a highly resolutive barcode, expanding the genus to include multiple newly recognized species [1, 2]. These ubiquitous organisms are found in plants, soil, water, and animal waste, particularly in tropical areas [3–5]. Comprehensive environmental screenings have demonstrated their widespread distribution in diverse aquatic ecosystems, with successful isolation from rivers, lakes, urban ponds, and agricultural wastewater [2]. Furthermore, targeted field studies have confirmed their high prevalence in anthropogenic habitats, particularly in liquid manure, cattle barn flush water, and sewage, highlighting their strong association with animal waste management systems [2, 6]. Under certain conditions, *Prototheca* spp. can act as opportunistic pathogens, causing various pathologies in both animals and humans. To date, six species within this genus have been explicitly recognized as pathogenic agents in vertebrates, namely, *Prototheca wickerhamii*, *Prototheca bovis*, *Prototheca ciferrii*, *Prototheca blaschkeae*, *Prototheca cutis*, and *Prototheca miyajii* [1, 2]. Among these, *P. wickerhamii* represents the primary etiological agent responsible for human protothecosis, typically manifesting as cutaneous, articular, or disseminated systemic infections, whereas *P. bovis* is widely documented as the principal cause of bovine mastitis, leading to substantial economic losses in the dairy sector globally [1, 2].

The first human case of protothecosis was reported by Davies, Spencer, and Wakelin in 1964 [7]. To date, more than 200 cases have been documented in medical literature, primarily attributed to *Prototheca zopfii* (historically encompassing pathogenic species recently reclassified as *P. bovis* and *P. ciferrii*, while *P. zopfii* sensu stricto is now considered a nonpathogenic environmental species [1] and *Prototheca wickerhamii* [8]). Clinically, protothecosis manifests mainly in three forms: skin and subcutaneous tissue infection, synovitis and fibrous tissue inflammation, and disseminated (systemic) infection. Cutaneous infection is the most common presentation, often observed in exposed areas like the extremities and face [9]. Lesions can appear as nodules, papules, verrucous lesions, plaques, herpetiform lesions, or ulcers, frequently linked to traumatic inoculation [10–12].

While cases of protothecosis have been reported in various Asian countries, including Japan, China [13], Thailand [14], and Singapore [14], reports of human protothecosis in Vietnam remain rare, with only a historical case of olecranon bursitis reported in an American soldier in South Vietnam in 1970 [15]. This report presents two confirmed Vietnamese cases of cutaneous protothecosis and explores the diagnostic and therapeutic challenges encountered.

## 2. Case Presentation

Case 1: A 63‐year‐old female ice picker presented with a 2‐month history of painful, nonhealing ulcers on both upper extremities. She reported a 20‐year history of continuous occupational exposure to potentially contaminated water; however, no preceding local skin trauma was reported. The lesions initially appeared as erythematous plaques, rapidly evolving into ulcerations. Her medical history was significant for hypertension, but she had no known immunosuppressive conditions.

Ten days prior to admission, she was evaluated at an outpatient clinic where a skin scraping was reported positive for “septate hyphae.” She was prescribed oral itraconazole 200 mg/day in combination with topical terbinafine, but her lesions continued to extend. One week later, she was seen by another physician at the outpatient clinic with a diagnosis of superficial fungal infection with bacterial superinfection. She received oral amoxicillin–clavulanic acid 1 g twice daily. However, her symptoms did not improve, and she experienced significant pain, even when raising her arm or untying bandages. Upon admission, she had completed a 10‐day course of systemic itraconazole and a 4‐day course of amoxicillin–clavulanic acid.

Physical examination revealed ulcers with hemorrhagic crusting and scabs over the extensor aspects of both arms (Figure 1A,B). Differential diagnoses considered included deep fungal infection and atypical mycobacterial infection. Laboratory tests, including peripheral blood cell count, urine analyses, liver and kidney function, and lymphocyte subpopulations, were all within normal ranges.

**FIGURE 1:**
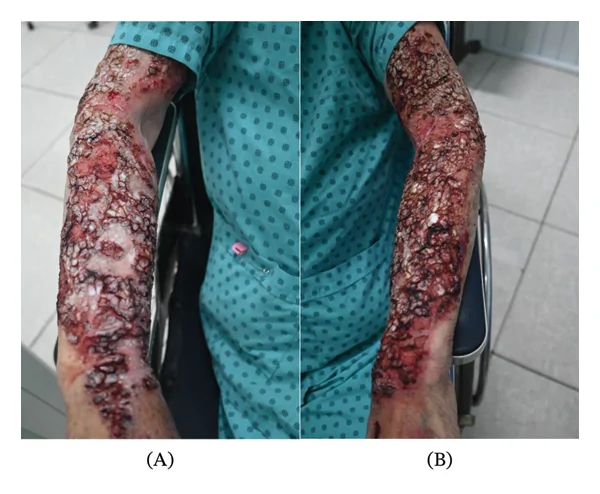
(A‐B) (Case 1): Well‐defined ulcers with scab over extensor surface of both arms (A: right arm, B: left arm).

Histopathological examination of a skin biopsy revealed necrotic epidermis, lymphohistiocytic infiltrate with sporangia in both the epidermis and dermis (Figure 2). Periodic acid‐Schiff (PAS) and Grocott–Gömöri methenamine silver (GMS) staining highlighted numerous PAS‐positive and GMS‐positive bodies, forming symmetrical morula or cartwheel‐like structures packed together amidst the inflammatory infiltrate and within the epidermis (Figures 3 and 4). For microbiological evaluation, the tissue biopsy specimen was macerated in Sabouraud broth (SAB broth) and incubated at 30°C–37°C for 16–24 h. Direct microscopic examination was performed, followed by subculture onto Chloramphenicol Yeast Glucose Agar (SAB‐Cl). Cultures were incubated simultaneously at two temperature conditions (20°C–25°C and 35°C–37°C) and monitored for up to 10 days. Despite these characteristic findings, fungal cultures of the lesion on Chloramphenicol Yeast Glucose Agar were negative. Based solely on the histopathological examination, a diagnosis of protothecosis was made. To rule out mimicking infectious etiologies, skin biopsy specimens were also tested via polymerase chain reaction (PCR) for *Mycobacterium tuberculosis* and nontuberculous mycobacteria (NTM), both yielding negative results. Bacterial culture of the tissue biopsy isolated *Klebsiella aerogenes*. Antimicrobial susceptibility testing demonstrated resistance to amikacin, amoxicillin/clavulanic acid, ampicillin, cefepime, cefotaxime, cefoxitin, ceftazidime, ceftriaxone, cefuroxime, ciprofloxacin, gentamicin, imipenem, piperacillin/tazobactam, and tobramycin. The patient was subsequently transferred to a specialized tertiary hospital for further evaluation of a suspected lung malignancy. During that admission, she underwent local surgical debridement and received a 14‐day course of intravenous imipenem along with standard wound care for suspected secondary bacterial superinfection. Interestingly, despite receiving no targeted antiprotothecal or systemic antifungal therapy, her cutaneous ulcers improved markedly. Given the in vitro resistance of the isolated *K. aerogenes* to imipenem, the clinical improvement suggested that *K. aerogenes* likely represented a secondary colonizer rather than the primary driver of the ulcerations. The patient was discharged following clinical improvement and was subsequently lost to long‐term follow‐up.

**FIGURE 2:**
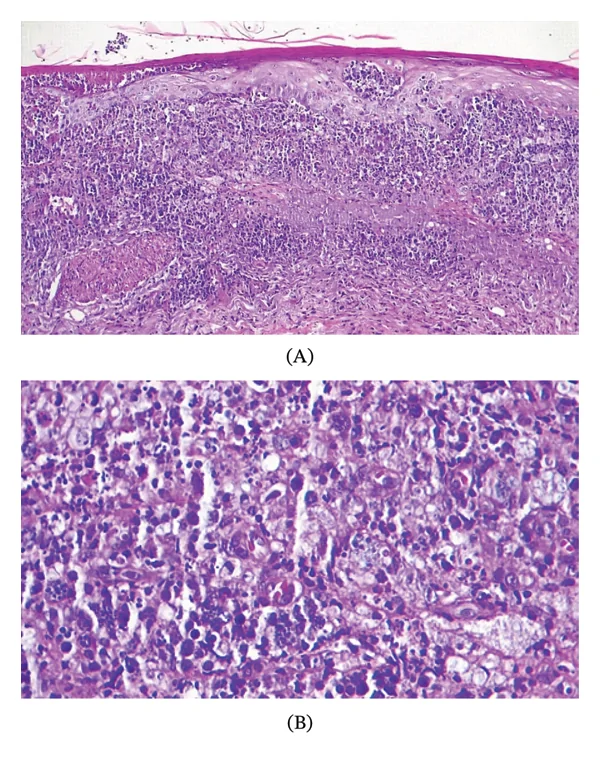
(Case 1): (A) Hematoxylin and eosin (H&E) stain showing necrotic epidermis and mixed inflammatory infiltrate (magnification × 100) and (B) H&E stain showing sporangia (magnification × 400).

**FIGURE 3:**
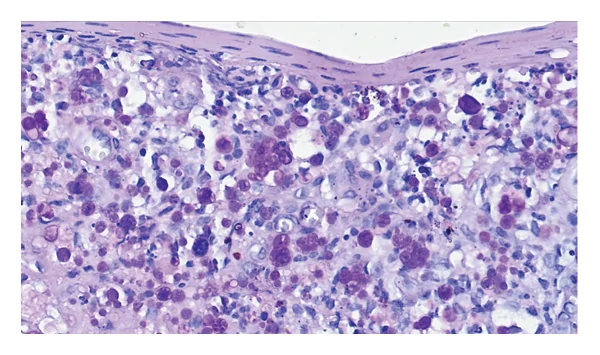
(Case 1): PAS stain demonstrating numerous *Prototheca* bodies forming cartwheel‐like structures (magnification × 400).

**FIGURE 4:**
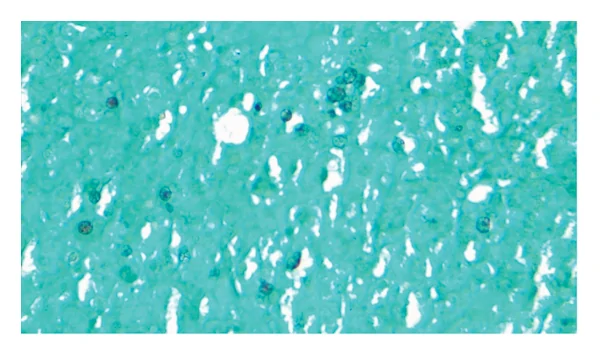
(Case 1): Characteristic thick‐walled *Prototheca* sporangia highlighted by GMS stain at × 400 magnification.

## 3. Case 2

An 81‐year‐old female presented with a 1‐month history of progressive, nonhealing ulcers on both forearms. Initially, a small ulcer appeared on her left hand, for which she received a diagnosis of bacterial infection and was treated with levofloxacin. Despite antibiotic therapy, the ulcer failed to improve and subsequently spread to involve both arms. Her medical history included chronic ischemic heart disease managed with rivaroxaban, hypertension, and an atrial septal defect for which she had undergone repair with a 30‐mm Cocoon device. She had no documented history of local trauma or occupational exposure.

Clinical examination revealed erythematous, eroded plaques with overlying superficial ulcers and necrotic crusts on the extensor aspects of both forearms. A skin biopsy was performed, which showed epidermal atrophy and a lymphohistiocytic infiltrate in the superficial and mid‐dermis (Figure 5A,B). Microscopic examination further identified diffuse spore‐like structures with internal septations characteristic of *Prototheca* (Figure 6). GMS and PAS staining highlighted numerous sporangia containing abundant endospores within the dermis, a finding highly suggestive of *Prototheca* species (Figures 7 and 8). These histopathological findings, correlated with the clinical presentation, led to a diagnosis of cutaneous protothecosis.

**FIGURE 5:**
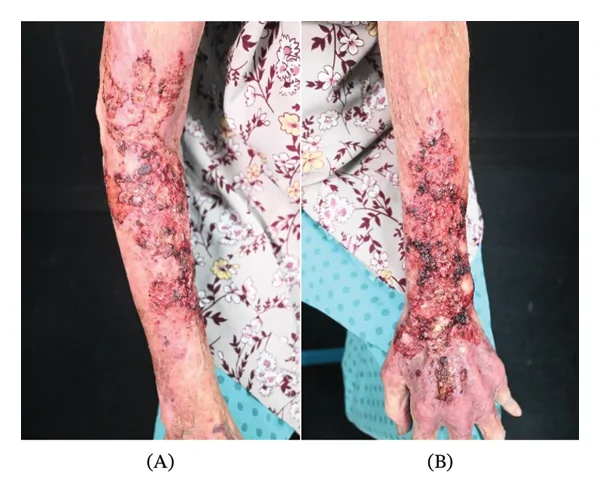
(A‐B) (Case 2): Well‐demarcated ulcers with overlying eschars on the extensor aspects of both upper extremities (A: right arm, B: left arm).

**FIGURE 6:**
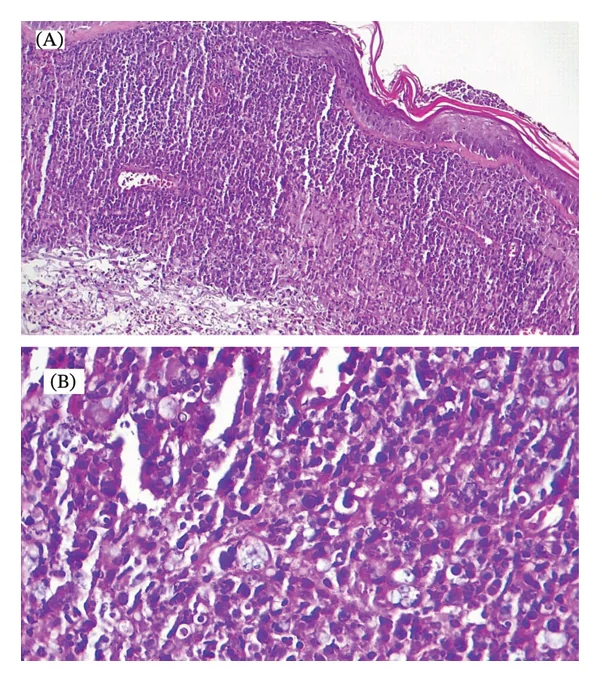
(A‐B) (Case 2): H&E stain showing atrophic epidermis and mixed inflammatory infiltrate with sporangia (magnification × 100 and × 400).

**FIGURE 7:**
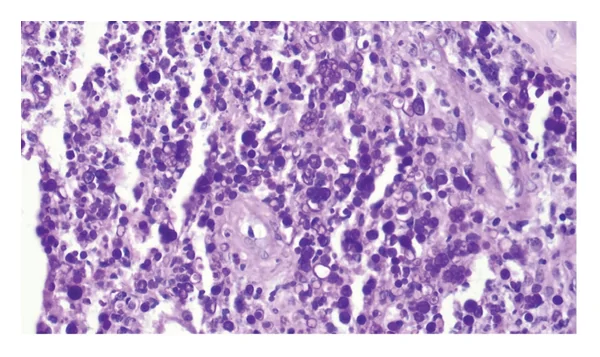
(Case 2): Periodic acid‐Schiff (PAS) stain reveals a high density of *Prototheca* organisms organized into cartwheel‐like structures (magnification × 400).

**FIGURE 8:**
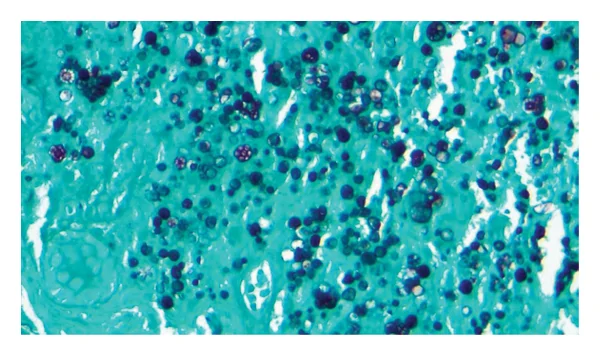
(Case 2): Microscopic view at × 400 magnification using a GMS stain to emphasize the distinctive thick walls of *Prototheca* sporangia.

Despite the strong histopathological evidence, fungal cultures on Chloramphenicol Yeast Glucose Agar repeatedly yielded negative results, utilizing the same protocol described in Case 1. PCR testing on the skin biopsy for *M. tuberculosis* and NTM was negative. Bacterial tissue culture isolated methicillin‐resistant *Staphylococcus aureus* (MRSA). Susceptibility testing indicated sensitivity to linezolid, tetracycline, and trimethoprim/sulfamethoxazole, but resistance to amoxicillin/clavulanic acid, cefoxitin, cefuroxime axetil, ciprofloxacin, clindamycin, erythromycin, gentamicin, and penicillin. The patient was treated with intravenous vancomycin for 10 days, resulting in significant clinical regression of the skin lesions. She was discharged home without systemic antifungal or antiprotothecal therapy and was subsequently lost to follow‐up.

## 4. Discussion

Protothecosis is a rare opportunistic infection, primarily affecting immunocompromised individuals. Its clinical presentation often mimics other infections, such as atypical mycobacterial and deep fungal infections, frequently leading to initial empirical treatments with antibacterial and antifungal agents. This diagnostic challenge was evident in our cases: the first patient was initially misdiagnosed with superficial dermatophytosis and received ineffective itraconazole and topical terbinafine, while the second patient’s initial diagnosis of a cutaneous bacterial infection led to unsuccessful antibiotic therapy. These instances emphasize the critical necessity for prompt histopathological examination and culture to achieve accurate diagnosis, particularly in the absence of a favorable clinical response to empirical therapeutic regimens.

Nevertheless, our two cases exhibited a consistent clinical pattern of erythematous plaques with overlying ulceration and necrotic crusts, specifically on the extensor surfaces of both forearms. This presentation was sufficiently distinctive that the inpatient physician, having encountered the first case, swiftly considered cutaneous protothecosis upon physical examination of the second patient 4 months later. While cutaneous protothecosis is known for its varied morphology, including nodules, papules, verrucous lesions, and herpetiform lesions, our findings emphasize that extensive ulcers with underlying plaques on the extensor surface of both forearms in an elderly patient should serve as a crucial clinical clue for prompt diagnosis of cutaneous protothecosis. Compared to previously reported cases, our cases exhibited similar clinical findings, including erythematous plaques and superficial ulcers on exposed areas. However, a notable distinction was the significantly more extensive and bilateral involvement of the upper extremities in our patients, contrasting with the predominantly unilateral presentation in most published reports (Table 1) [16–20]. This could be attributed to either simultaneous invasion of *Prototheca* through minor skin injuries on both arms or the possibility of autoinoculation, where the infection spread from one arm to the other, possibly through scratching or direct contact by the patient.

**TABLE 1:** Comparison of the present cases with previously reported cases of cutaneous protothecosis in the literature (1994–2024).

Characteristic	Present cases (*n* = 2)	Previously reported cases (*n* = 71; 45 publications)
Age, years	63, 81 (both female)	Mean 66.8; range 17–86
Sex (M:F)	0:2	39:32 (55%:45%)
Immune status	Immunocompetent (2/2, 100%)	Immunocompromised 47/71 (66%); immunocompetent 24/71 (34%)
Culture‐confirmed species	Negative in both cases; diagnosis by histopathology alone	*P. wickerhamii* 38/71 (54%); *P. zopfii* (incl. variants) 3/71 (4%); not confirmed by culture 29/71 (41%)
Distribution of lesions	Bilateral, both forearms (2/2, 100%)	Bilateral involvement in 3/71 (4%); predominantly unilateral/single‐site in the remainder
Predominant morphology	Extensive erythematous plaques with ulceration and necrotic crusts	Nodules, papules, plaques, verrucous lesions, or ulcers (variable)
Treatment	Imipenem, surgical debridement and standard wound care (Case 1); vancomycin, surgical debridement and standard wound care (Case 2)	Azole antifungals (itraconazole, fluconazole, voriconazole) most common; amphotericin B used for severe/disseminated disease
Outcome	Improved (2/2, 100%)	Cured/improved 61/71 (86%); died 7/71 (10%); lost to follow‐up or not reported 3/71 (4%)

(This study compiles data from 45 previously published case reports and case series on cutaneous protothecosis from 1994 to 2024. A complete listing containing individual patient data and references is provided in Supporting Table S1).

In our cases, definitive identification rested on histopathological examination due to negative culture results. Standard microbiological workup for tissue biopsies involved enrichment in Sabouraud broth, followed by isolation on chloramphenicol‐supplemented agar, incubated at both 20°C–25°C and 35°C–37°C for up to 10 days. Although fungal culture is considered the microbiological reference standard for diagnosing protothecosis, negative culture results do not exclude the diagnosis. In our cases, several factors may have contributed to culture negativity, including prior antimicrobial exposure, reduced organism viability, limited tissue sampling, or suboptimal culture conditions. The use of povidone–iodine before biopsy may also have played a role, given its reported algicidal activity against *Prototheca* species. Although molecular techniques such as PCR sequencing of rRNA genes or MALDI‐TOF MS provide rapid and definitive species identification, these diagnostic modalities were unavailable in our laboratory setting at the time, highlighting the crucial reliance on characteristic histopathology (PAS and GMS positive sporangia with internal endospores) in resource‐limited or culture‐negative settings.

The lack of response to itraconazole in Case 1 is noteworthy. While this might raise suspicion of a poor response to this specific azole, a definitive conclusion regarding itraconazole resistance cannot be drawn due to the short duration of treatment, the absence of a positive culture, and the lack of in vitro susceptibility testing. Other factors, such as the compounding presence of a bacterial superinfection, could also account for the initial treatment failure. Treatment of protothecosis primarily relies on an empirical approach, guided by in vitro susceptibility testing (MIC cultures). Unfortunately, this was unavailable in our case due to the negative fungal culture. Given the first patient’s nonresponse to itraconazole, newer azole antifungals like voriconazole could be considered for future management.

Generally, disseminated infections typically require treatment with amphotericin B, whereas localized infections are usually managed with azole antifungals [8]. In vitro drug susceptibility profiling on isolates from human cases of protothecosis often shows good activity with amphotericin B and most azoles, exhibiting an algicidal rather than algistatic effect against *Prototheca* [21]. However, a recent review of protothecosis infections in China reported itraconazole as the most successful single treatment, followed by fluconazole, ketoconazole, amphotericin B, and voriconazole [22]. This highlights the variability in treatment responses and the ongoing need for clearer guidelines.

This report has several limitations. The diagnosis of protothecosis in both cases relied solely on histopathological identification of characteristic PAS‐ and GMS‐positive morula‐like sporangia, as fungal cultures were repeatedly negative. Culture negativity may have resulted from prior antimicrobial exposure, reduced organism viability, limited tissue sampling, suboptimal culture conditions, or the prebiopsy application of povidone–iodine, which possesses reported algicidal activity against *Prototheca* species. Nonetheless, negative cultures do not rule out protothecosis, and characteristic histopathological features remained essential for establishing the diagnosis. Without viable isolates, species identification (e.g., *Prototheca wickerhamii* vs. *P. zopfii*) via MALDI‐TOF MS or molecular sequencing, as well as in vitro antifungal susceptibility testing, could not be performed. Consequently, treatment considerations relied on published literature rather than isolate‐specific data. Finally, both patients were lost to long‐term follow‐up, precluding definitive evaluation of treatment outcomes.

## Funding

No funding was received for this manuscript.

## Conflicts of Interest

The authors declare no conflicts of interest.

## Supporting Information

Additional supporting information can be found online in the Supporting Information section.

## Supporting information


**Supporting Information** Supporting Table S1: Previously Reported Cases Of Cutaneous Protothecosis In The Literature (1994–2024).

## Data Availability

The clinical data used to support the findings of this study are available from the corresponding author upon reasonable request.
