# Supplementary material for: Cutaneous Protothecosis: Reported Human Cases in Vietnam and Diagnostic Challenges
Source: Case Rep Dermatol Med. 2026 Oct 9;2026:9136305. doi: 10.1155/crdm/9136305 (PMC13651540; doi:10.1155/crdm/9136305)
Supplement: Supplementary file 1 [file CRDM-2026-9136305-s001.docx]

**Supplementary Table S1:** **Previously Reported Cases Of Cutaneous Protothecosis In The Literature (1994–2024)**

| **Patients** | **Year** | **Age** | **Sex** | Country | **Underlying condition** | **Immunity status** | **Initial symptoms** | Species | **Coinfection** | **Treatment** | **Outcome** | **Reference** |
| --- | --- | --- | --- | --- | --- | --- | --- | --- | --- | --- | --- | --- |
| 1 | 1994 | 33 | woman | USA | AIDS, pulmonary TB, Pneumocystis carinii pneumonia, primary cutaneous B-cell lymphoma | Severely immunocompromised | Erythematous-yellow verrucous, crusted plaque on dorsum of right hand | P. wickerhamii | Pulmonary TB, PCP, MAC, cerebral toxoplasmosis | Intravenous amphotericin B | Died (<24h after treatment) | [1] |
| 2 | 1995 | 70 | man | Hong Kong | Left knee arthritis (pseudogout), chronic hepatitis B | Immunocompetent | Erythematous ulcerated plaque on left knee, spreading over 12 months; later scaly papules on legs, face, forearm | P. wickerhamii | Candida albicans (secondary wound colonization) | Oral itraconazole 200 mg/day + surgical debridement | Improved, not fully healed | [2] |
| 3 | 1995 | 20 | man | Italy (Somali patient) | Unclear; decreased CD4/CD8 ratio (0.8), HIV negative | Mildly immunocompromised | Erythematous nodule on right knee after trauma, progressing to central necrotic ulcer | P. wickerhamii | None reported | Tioconazole + itraconazole 200 mg/day + doxycycline 100 mg/day | Marked improvement after 1 month of treatment | [3] |
| 4 | 1997 | 39 | man | USA | AIDS-related lymphoma, chemotherapy-induced neutropenia, S. aureus bacteremia | Severely immunocompromised (CD4 3/mm3, neutrophil oxidative burst defect) | Pruritic hypopigmented, crusted papules on nose and face (3-month history) | P. wickerhamii | Staphylococcus aureus bacteremia | Itraconazole (failed) switched to intravenous amphotericin B | Cured (healed after 2 weeks) | [4] |
| 5 | 2001 | 72 | woman | Brazil | None (immunocompetent); prolonged topical clobetasol use | Immunocompetent | Erythematous infiltrated plaques on flexor right arm/forearm after trauma (abrasion) | P. wickerhamii | None reported | Fluconazole 150 mg/day × 30 days, then 150 mg/week × 60 days | Complete resolution, no relapse at 18 months | [5] |
| 6 | 2002 | 77 | man | Taiwan | Diabetes mellitus, untreated; farmer | Immunocompromised (uncontrolled DM) | Pyoderma-like erythematous plaque with pustules and ulcers on right leg (2 weeks) | P. wickerhamii | None reported | IV amphotericin B + oral tetracycline × 2 weeks | Cured | [6] |
| 7 | 2002 | 71 | man | Taiwan | Gouty arthritis, steroid abuse with Cushing syndrome, topical potent steroid; farmer | Immunocompromised (systemic/topical steroid) | Erythematous scaling plaque with scattered papules on left forearm (1 month) | P. wickerhamii | None reported | Intralesional amphotericin B × 3 weeks | Cured | [6] |
| 8 | 2002 | 69 | man | Taiwan | Frequent IV injections, bronchial asthma, gouty arthritis, long-term steroid use; farmer | Immunocompromised (long-term steroid use) | Diffuse erythema/scaling on forearms and hands with infiltrating nodules (several weeks) | P. wickerhamii | None reported | Itraconazole × 3 weeks (failed) → fluconazole + topical amphotericin B wet dressing | Died (cardiopulmonary insufficiency from asthma, unrelated to protothecosis) | [6] |
| 9 | 2002 | 67 | woman | Taiwan | Pesticide exposure, diabetes mellitus, iatrogenic Cushing syndrome; farmer | Immunocompromised (DM, iatrogenic Cushing syndrome) | Painful pyoderma-like papules/plaques with ulcers, linear distribution on right leg (10 days) | P. wickerhamii | None reported | Fluconazole × 3 weeks + surgical debridement | Cured | [6] |
| 10 | 2002 | 43 | woman | Taiwan | Prolonged topical steroid use; labourer | Immunocompromised (topical steroid) | Painful erythematous plaque with telangiectasia on nose (3–4 months) | Not confirmed by culture (histology only) | None reported | Ketoconazole × 7 weeks | Cured | [6] |
| 11 | 2004 | 59 | woman | Brazil | None; blunt trauma to finger, local steroid infiltration, soil/gardening exposure | Immunocompetent | Chronic inflammatory lesion on right 4th finger (edema, hyperemia, purulent discharge) after trauma, worsening over ~3 years | P. wickerhamii | Staphylococcus aureus (secondary infection) | Itraconazole 400 mg/day × 6 weeks (failed) switched to fluconazole 200 mg/day | Gradual healing, improving at 6-month follow-up | [7] |
| 12 | 2006 | 70 | woman | Brazil | None (healthy); topical corticosteroid use; gardening hobby | Immunocompetent | Pruriginous, progressively thickening eczema-like plaque with pustules on right forearm (1 year) | P. wickerhamii | None reported | Itraconazole 200 mg/day × 30 days, then 100 mg/day × 60 days | Complete regression, no relapse at 6 months | [8] |
| 13 | 2009 | 77 | man | Canada | Osteoarthritis, hypertension, hyperlipidemia; tape-stripping injury post-cataract surgery; recreational hot tub use | Immunocompetent (no immunosuppressive medication) | Progressively enlarging plaques on left cheek and supraclavicular fossa (1 year) at injury site | Prototheca sp. (species not identified) | None reported | Oral fluconazole 100 mg/day × 6 weeks (recurred at 3 months) + additional 11-week course | Cured, no recurrence at 5 years | [9] |
| 14 | 2010 | 78 | man | Japan | Nephrosis on long-term prednisolone 30 mg/day; minor trauma | Immunocompromised (long-term corticosteroid) | Erythematous plaques with pustules, crusts and ulcers on dorsal right hand/forearm (1 week) | P. wickerhamii | None reported | Itraconazole 100→400 mg/day + topical ketoconazole (failed) → added local thermal therapy (46°C) + itraconazole 400 mg/day | Complete resolution after 2 months, no recurrence >2 years | [10] |
| 15 | 2024 | 82 | man | China | Hypertension; stab wound to left forearm; topical clobetasol aggravated lesion | Immunocompetent | Diffuse infiltrated erythematous/indurated plaques with insect-bite-like papules, ulcers, pustules on dorsal left forearm/hand (4 months) | P. wickerhamii | None reported | Itraconazole 200 mg/day + topical naftifine-ketoconazole × 4 weeks (GI side effects) → switched to terbinafine 250 mg/day + topical cream × 3 months | Complete healing (fibrotic scar), no relapse at 1.5 years | [11] |
| 16 | 2012 | 66 | woman | China | Diabetes mellitus (10 y), hypertension (20 y); non-healing post-surgical wound on finger | Immunocompromised (diabetes) | Non-healing surgical wound with palpable nodules on right middle finger, restricted movement (8 months) | P. zopfii var. portoricensis | None reported | IV amphotericin B 15→25 mg/day × 23 days + surgical debridement + itraconazole (IV then oral) × 3 months | Improved (reduced abscesses, better flexion), residual scarring/tendon damage | [12] |
| 17 | 2012 | 74 | woman | Thailand | None; topical steroid and herbal paste use | Immunocompetent | Erythematous pruritic patches on lower abdominal wall (4 months), no trauma history | P. wickerhamii | None reported | Itraconazole 200 mg/day (stopped after 1 week, GI side effects) → doxycycline 200 mg/day × 3 months | Cleared, no recurrence at 6 months | [13] |
| 18 | 2012 | 69 | man | China | Diabetes mellitus (untreated, 6 months); traumatic injury | Immunocompromised (uncontrolled DM) | Erythematous plaques with vesiculobullous crust on dorsal right forearm/hand/leg (6 months), severe pain | P. wickerhamii | Aeromonas hydrophila (necrotizing fasciitis) | IV voriconazole + cephalosporin × 14 days → oral antifungal × 14 days (incomplete, self-discharged) | Died (septicemia, weeks later) | [14] |
| 19 | 2012 | 44 | woman | China | Psoriasis (10 years), long-term topical steroid use | Immunocompromised (chronic topical steroid) | Edema with ulcerative, crusted lesion on abdomen, fever (10 days) | P. wickerhamii | None reported | IV fluconazole (moderate improvement) → switched to IV then oral voriconazole | Significant improvement, no organism on follow-up culture | [14] |
| 20 | 2013 | 61 | woman | Brazil | Hypertension, allergic rhinitis on chronic systemic corticosteroids, new type II DM and neuropathy; trauma during hospitalization | Immunocompromised (chronic systemic corticosteroid use) | Erythematous, painful plaque with pustules and scabs on right leg (15 days) | P. wickerhamii | None reported | Itraconazole 200 mg/day × 3 months | Complete healing | [15] |
| 21 | 2013 | 61 | man | Australia | Hypogammaglobulinemia (likely CVID); extensive travel/scuba diving exposure | Immunocompromised (humoral immunodeficiency) | Multiple recurrent ulceronodular skin lesions on exposed extremities (3 years) | P. wickerhamii | None reported | Multiple prior excisions; itraconazole (failed) → oral voriconazole 400 mg bid loading, then 200 mg bid × 3 months | Resolution, no relapse at 6 months | [16] |
| 22 | 2013 | 68 | man | South Korea | Osteoarthritis (20 y); prior bowel surgery; occupation as trash collector | Immunocompetent | Ill-defined erythematous nodular lesion with crust on left wrist (1 month), no trauma history | P. zopfii (rare) | None reported | Itraconazole | Symptoms relieved (improved) | [17] |
| 23 | 2015 | 58 | man | Singapore | Chronic psoriasiform rash/prurigo nodularis on topical steroids; newly diagnosed HIV (CD4 38/µL); shaving cut | Immunocompromised (previously undiagnosed HIV) | Gradually enlarging erythematous scaly plaque on left cheek (6 weeks) | P. wickerhamii (presumptive, morphology) | HIV infection (newly diagnosed) | Oral itraconazole 200 mg/day + antiretroviral therapy | Significant improvement at 2 weeks | [18] |
| 24 | 2015 | 70 | woman | South Korea | Hypertension, diabetes mellitus (2 y); past pulmonary tuberculosis (resolved) | Immunocompromised (diabetes) | Itchy erythematous plaques with exudate on both forearms (2 months) | P. wickerhamii (confirmed by PCR on paraffin tissue) | None reported | Itraconazole 200 mg/day × 6 weeks (failed) → voriconazole 400 mg/day × 2 months → itraconazole 200 mg/day × 5 months maintenance | Complete resolution (atrophic scars), no relapse at 1 year | [19] |
| 25 | 2016 | 74 | woman | South Korea | None; long-term topical corticosteroid use (2 y); pet dog exposure (18 y) | Immunocompetent (local immunosuppression from topical steroid) | Erythematous umbilicated papules on anterior neck/upper chest (2 months) on pre-existing telangiectatic patch | P. wickerhamii (morphologic) | None reported | Oral itraconazole 200 mg/day + topical sertaconazole × 12 weeks | Marked improvement (fibrotic scar) | [20] |
| 26 | 2015 | 72 | woman | USA | Bilateral mastectomy (breast cancer), insulin-dependent DM, hypertension, CHF, chronic lymphocytic leukemia on chemotherapy (cyclophosphamide/vincristine/prednisone) | Severely immunocompromised (CLL + chemotherapy) | Pustular and erythematous plaques on dorsal upper extremities (3 months) | P. wickerhamii | None (concurrent leukemic skin infiltrate) | Oral tetracycline + IV amphotericin B | Died from other comorbidities before treatment could succeed | [21] |
| 27 | 2015 | 80 | man | China | None; immunocompetent, no trauma history | Immunocompetent | Erythematous plaques, dark red papules, superficial ulcerations on right chest/neck mimicking eczema (3 months) | P. wickerhamii (confirmed by culture and molecular sequencing) | None reported | Itraconazole 200 mg/day + topical ketoconazole 2% cream | Cured (subsided at 4 weeks, residual pigmentation) | [22] |
| 28 | 2016 | 63 | man | Australia | Dermatomyositis with pulmonary fibrosis, T2DM, peripheral neuropathy, CKD stage III, CHF, atrial fibrillation, primary biliary cirrhosis; prior mycobacterial infections; long-term prednisolone 30 mg/day; frequent hot-tub use | Immunocompromised (chronic corticosteroid, multiple comorbidities) | Right lower limb swelling, erythema and ulceration with bullae (2 weeks) | P. wickerhamii | MRSA (biopsy PCR-positive) | IV liposomal amphotericin B (stopped for renal decline) → oral itraconazole 200 mg bid | Healed by 6 months, minor residual granulation | [23] |
| 29 | 2017 | 72 | woman | Singapore | Hypertension, hyperlipidemia; long-term topical/injected corticosteroids for presumed eczema; occupation as food handler (25 y, raw fish/seafood without gloves) | Immunocompetent (local immunosuppression from corticosteroids) | Bilateral dorsal forearm erythematous plaques with pustules (1 year) | Not confirmed by culture (histology only) | None reported | Itraconazole 100 mg bid | Marked improvement in 1 month, complete resolution | [24] |
| 30 | 2017 | 85 | woman | Japan | None; topical clobetasol use, no trauma history | Immunocompetent | Infiltrative erythema and ulceration with moth-eaten appearance on right forearm | P. wickerhamii genotype 2 | None reported | Fluconazole 100 mg/day × 14 days (failed, renal dysfunction) → itraconazole 200 mg/day × 2 months | Resolved, no recurrence at 2 years | [25] |
| 31 | 2018 | 71 | woman | Taiwan | Rheumatoid arthritis, COPD; methylprednisolone 6 mg/day × 2 y (iatrogenic Cushing syndrome) | Immunocompromised (chronic corticosteroid) | Erythematous plaque with pustules and punctate ulcers on limb | Not identified (no viable culture) | None reported | Itraconazole × 14 days | Improved | [26] |
| 32 | 2018 | 74 | man | Taiwan | Iatrogenic Cushing syndrome (black pills); trauma (pipe) | Immunocompromised (iatrogenic Cushing syndrome) | Erythematous plaque with pustules and punctate ulcers on limb | Not identified | Scabies (Sarcoptes scabiei) | Itraconazole × 28 days (persisted) → amphotericin B × 10 days | Improved but intolerable adverse effects | [26] |
| 33 | 2018 | 69 | woman | Taiwan | Diabetes mellitus; iatrogenic Cushing syndrome (black pills) | Immunocompromised (DM, iatrogenic Cushing syndrome) | Erythematous plaque with pustules and punctate ulcers on limb | Not identified | Scabies; MRSA sepsis (fatal, unrelated to protothecosis) | Amphotericin B × 21 days (630 mg cumulative) | Skin lesion improved, died of MRSA sepsis | [26] |
| 34 | 2018 | 74 | woman | Taiwan | Diabetes mellitus; iatrogenic Cushing syndrome (black pills); trauma (bamboo) | Immunocompromised (DM, iatrogenic Cushing syndrome) | Erythematous plaque with pustules and punctate ulcers on limb | Not identified | Scabies | Itraconazole × 14 days | Improved | [26] |
| 35 | 2018 | 69 | woman | Taiwan | Diabetes mellitus, asthma, osteoarthritis both knees; methylprednisolone 4-12 mg/day × 5 y; trauma (venipuncture) | Immunocompromised (DM, chronic corticosteroid) | Erythematous plaque with pustules and punctate ulcers on limb | Not identified | None reported | Itraconazole × 28 days | Improved | [26] |
| 36 | 2018 | 48 | man | Taiwan | Spondylolisthesis L4/5; iatrogenic Cushing syndrome (black pills) | Immunocompromised (iatrogenic Cushing syndrome) | Erythematous plaque with pustules and punctate ulcers on limb | Not identified | None reported | Itraconazole × 14 days | Improved | [26] |
| 37 | 2018 | 65 | man | Taiwan | History of alcohol abuse; no steroid use | Immunocompetent | Erythematous plaque with pustules and punctate ulcers on limb | Not identified | None reported | Itraconazole × 21 days | Improved | [26] |
| 38 | 2018 | 76 | woman | Taiwan | Osteoporosis with compression fractures; black pills, cortisone acetate 25 mg/day (iatrogenic Cushing syndrome) | Immunocompromised (iatrogenic Cushing syndrome) | Erythematous plaque with pustules and punctate ulcers on limb | Not identified | None reported | Itraconazole × 90 days | Improved | [26] |
| 39 | 2018 | 83 | woman | Taiwan | Vulvar carcinoma s/p surgery (20 y prior); trauma (fall) | Immunocompetent | Ulcerative erythematous papuloplaques on right hand | P. wickerhamii | None reported | Itraconazole × 28 days | Improved | [26] |
| 40 | 2018 | 76 | man | Taiwan | Diabetes mellitus; businessman | Immunocompromised (DM) | Erythematous plaque with pustules and punctate ulcers on limb | Not identified | None reported | Itraconazole × 14 days | Improved | [26] |
| 41 | 2018 | 82 | woman | Taiwan | Diabetes mellitus, spondylolisthesis L4/5; cortisone acetate 25 mg/day × 10 y (iatrogenic Cushing syndrome) | Immunocompromised (DM, iatrogenic Cushing syndrome) | Erythematous plaque with pustules and punctate ulcers on limb | P. wickerhamii | Scabies | Itraconazole × 98 days (recurrence) → retreated × 48 days | Recurrent, then improved | [26] |
| 42 | 2018 | 85 | man | Taiwan | Diabetes mellitus, gout, spondylolisthesis L4/5; prednisolone 10 mg/day × 3 y (iatrogenic Cushing syndrome); farmer | Immunocompromised (DM, iatrogenic Cushing syndrome) | Ulcerative erythematous papuloplaques on right hand | Not identified | None reported | Itraconazole × 14 days | Improved | [26] |
| 43 | 2018 | 67 | woman | Taiwan | Diabetes mellitus; black pills, cortisone acetate (iatrogenic Cushing syndrome); trauma (fall) | Immunocompromised (DM, iatrogenic Cushing syndrome) | Infiltrative erythematous plaques on limb | Not identified | None reported | Itraconazole × 63 days (partial) then × 102 days more | Partial improvement, then improved | [26] |
| 44 | 2018 | 78 | man | Taiwan | Warthin tumour s/p surgery (7 y prior), gout, spondylolisthesis L4/5; prednisolone 30 mg/day × 2 months for eczema | Immunocompromised (corticosteroid) | Erythematous plaque with pustules and punctate ulcers on limb | Not identified | None reported | Itraconazole × 148 days | Improved | [26] |
| 45 | 2018 | 86 | woman | Taiwan | Herniated intervertebral disc s/p surgery; black pills (iatrogenic Cushing syndrome); construction worker | Immunocompromised (iatrogenic Cushing syndrome) | Geographic ulcers with erythematous plaques (suspected disseminated; blood culture negative) | Not identified | None reported | Itraconazole × 46 days | Skin lesion improved, died of sepsis (unrelated cause) | [26] |
| 46 | 2018 | 78 | man | Taiwan | Chinese herbal drugs of unknown content; construction worker | Immunocompromised (possible undisclosed steroid) | Erythematous plaque with pustules and punctate ulcers on limb | Not identified | None reported | Itraconazole × 49 days | Improved | [26] |
| 47 | 2018 | 77 | man | Taiwan | Gout; cortisone acetate 75 mg/day × 5 months (iatrogenic Cushing syndrome); farmer | Immunocompromised (iatrogenic Cushing syndrome) | Erythematous subcutaneous noduloplaques on left forearm | Not identified | None reported | Itraconazole × 148 days | Improved | [26] |
| 48 | 2018 | 84 | man | Taiwan | COPD; retired; trauma (venipuncture) | Immunocompromised (COPD) | Erythematous plaque with pustules and punctate ulcers on limb | Not identified | None reported | Itraconazole × 49 days | Improved | [26] |
| 49 | 2018 | 72 | man | Taiwan | COPD, gout, hepatocellular carcinoma s/p radiofrequency ablation; cortisone acetate 40 mg/day × 3 months; driver | Immunocompromised (COPD, corticosteroid) | Erythematous to purpuric plaques on left forearm with peripheral whitish scars | P. wickerhamii | None reported | Itraconazole × 28 days | Improved | [26] |
| 50 | 2018 | 67 | man | Taiwan | Cotton factory worker; trauma (fall) | Immunocompetent | Erythematous plaque with pustules and punctate ulcers on limb | Not identified | None reported | Itraconazole × 147 days | Improved | [26] |
| 51 | 2018 | 70 | man | USA | Type 2 diabetes mellitus (well controlled), psoriasis on ustekinumab 45 mg SC q12wks; no trauma/water exposure | Immunocompromised (IL-12/23 inhibitor therapy) | Painful, itchy erythematous patch with indurated papules on right forearm (1 month) | P. wickerhamii | None reported | Itraconazole 200 mg tid × 3 days, then 200 mg bid, total 2 weeks | Resolution of symptoms | [27] |
| 52 | 2018 | 72 | man | China | Hypoalbuminemia (20-year strict vegetarian diet); trauma (branch scrape, healed) then chronic itchy rash misdiagnosed as eczema | Immunocompromised (hypoalbuminemia) | Erythematous plaques with pustules, superficial ulcers, necrotic crusts on right forearm/elbow/upper arm (1 year) | P. wickerhamii | None reported | IV fluconazole 400 mg/day × 1 week → oral fluconazole 100 mg bid × 3 weeks maintenance (~2 months total) | Remarkable improvement, scarring, no recurrence at 6 months | [28] |
| 53 | 2018 | 34 | woman | China | None; no trauma history | Immunocompetent | Unilateral facial erythematous plaque with scattered papules, slowly spreading (5 years), mild pruritus | P. zopfii | None reported | Not reported | Not reported | [29] |
| 54 | 2019 | 77 | woman | Egypt | Remote (10 y) and recent dermal filler/autologous fat transfer injections to cheeks; no systemic disease reported | Immunocompetent | Bilateral tender, swollen, erythematous, crusted cheeks with purulent/necrotic discharge (1 month after corrective filler injection) | Not confirmed by culture (morphology only) | None reported | Surgical drainage + itraconazole 200 mg/day × 6 months | Marked improvement | [30] |
| 55 | 2020 | 84 | man | China | Type 2 diabetes mellitus (4 y, on insulin), gout (6 y); farmer | Immunocompromised (diabetes) | Skin edema progressing to multi-site necrotic ulcers (right ankle then right arm) over 3 months, initially misdiagnosed as bacterial soft-tissue infection | P. wickerhamii | None reported | IV fluconazole 200 mg q12h + topical econazole nitrate cream after debridement | Recovered within 2 weeks, fully recovered at 1 month | [31] |
| 56 | 2020 | 42 | man | Australia | None; recent military deployment to Iraq (possible water exposure) | Immunocompetent | Left anterior thigh erythematous papule progressing to crusted non-healing plaque (5 months) | Not confirmed by culture (histology only) | None reported | Itraconazole 100 mg bid + local heat therapy (54°C) → excision biopsy, itraconazole continued then ceased | Resolved (scar only) | [32] |
| 57 | 2020 | 69 | man | India | None; farmer, soil/plant contact with bare hands, recurrent minor trauma | Immunocompetent | Asymptomatic reticulate hyperpigmented plaque with atrophy on right forearm (3 months), incidental finding | Not confirmed by culture (morphology suggestive of P. wickerhamii) | None reported | Itraconazole 200 mg bid × 3 months | Significant improvement, lost to follow-up | [33] |
| 58 | 2020 | 60 | man | Brazil | Kidney transplant recipient (15 y), prior multiple squamous cell carcinomas; prednisone 5 mg/day + sirolimus 1 mg/day (5 y) | Immunocompromised (chronic transplant immunosuppression) | Erythematous nodule on right leg (6 months), no trauma history | Not confirmed by culture (histology only) | None reported | Complete surgical excision + secondary-intention healing (mupirocin, silver nitrate) + oral fluconazole 150 mg/day × 3 months | Complete healing, no relapse at 4 years | [34] |
| 59 | 2021 | 17 | man | China | None; full-body tattoo (2019); initially misdiagnosed as eczema/herpetic dermatitis | Immunocompetent | Wheal-like rash progressing to widespread erosions/ulcers (face, neck, trunk, limbs) with fever, followed by headache, nausea, vomiting (meningitis) | P. wickerhamii (first report isolated from both skin and CSF) | Staphylococcus (blood agar colonies, significance unclear) | Liposomal amphotericin B (reduced for toxicity) + fluconazole → switched to voriconazole (loading then maintenance) | Skin healed and neurological symptoms significantly improved after 2 months | [35] |
| 60 | 2022 | 66 | woman | India | Uncontrolled diabetes mellitus (HbA1c 14%), decreased CD3/CD4/CD8 T-cells; fisherwoman, recurrent trauma cleaning fish | Immunocompromised (diabetes, cellular immunodeficiency) | Painful erythematous edematous plaque with scarring/ulcers on right forearm and hand, mild fever (1 month) | Not confirmed by culture (histology only) | None reported | Amphotericin B 30 mg IV daily × 23 days → itraconazole 200 mg bid × 2 months | Healed with scarring after 2 weeks | [36] |
| 61 | 2022 | 65 | man | Brazil | Vitiligo; gardening, fishing trip to lagoon 5 y prior; misdiagnosed and treated as sporotrichosis | Immunocompetent | Erythematous papules on right arm (2 years) progressing to plaques, nodules, ulcerated tumoral mass with arm deformation and olecranon bursitis | Not identified to species (culture negative twice) | None reported | Itraconazole 200 mg/day × 12 months (failed) → surgical debridement (incl. olecranon bursa) + amphotericin B on alternate days (total 2.8 g) | Improved after debridement and amphotericin B | [37] |
| 62 | 2023 | 35 | woman | Argentina | Cretinism, chronic kidney disease, kidney transplant ×2 (graft rejection); prednisone, tacrolimus, mycophenolate mofetil | Severely immunocompromised (transplant immunosuppression) | Erythematous edematous plaque with diffuse borders on left leg, found incidentally during admission for urosepsis | Prototheca spp. (not further speciated) | Carbapenemase-producing Klebsiella pneumoniae (urosepsis) | Liposomal amphotericin B (improved) → itraconazole (partial/intermittent worsening) → fluconazole + minocycline at 9 months | Died of septic shock/multi-organ failure (Klebsiella urosepsis) | [38] |
| 63 | 2023 | 75 | man | Brazil | None reported; sewage exposure repairing clay pipe, minor arm trauma; initially misdiagnosed as dermatophytosis | Immunocompetent | Itchy hyperemic lesion progressing to painful infiltrative plaque spreading along entire left arm (6 months) | P. wickerhamii (confirmed by MALDI-TOF and ITS sequencing) | Secondary bacterial infection with mild discharge (organism not specified) | Itraconazole 400 mg/day (failed, progressed) → liposomal amphotericin B 4 mg/kg/day × 45 days → voriconazole 400 mg/day × 6 months | Complete healing, no recurrence at 18 months | [39] |
| 64 | 2023 | 40 | man | Brazil | Reactive arthritis on methotrexate 7.5 mg/week (1.5 y); aquarist, trauma washing aquarium algae | Immunocompromised (methotrexate) | Nodule on finger with purulent exudate, unresponsive to antibiotics | Not confirmed by culture (histology only) | None reported | Surgical excision + itraconazole 200 mg/day × 3 months | Almost complete regression | [40] |
| 65 | 2023 | 68 | man | Brazil | Hypertension, abdominal aortic aneurysm with obstructed vascular prosthesis; fisherman/canoeing (water exposure) | Immunocompromised (local vascular insufficiency) | Vesicles at penis base progressing to painful infiltrated erythema of inguinal region (6 months), unresponsive to antibiotics/acyclovir | Not confirmed by culture (histology only) | None reported | Itraconazole 400 mg/day × 6 months + aortic revascularization surgery | Total improvement | [40] |
| 66 | 2023 | 81 | woman | Australia | None; regular gardening; self-applied topical betamethasone | Immunocompetent | Painless erythematous nodules on right proximal forearm (3 years), slowly spreading to cubital fossa/distal upper arm | P. wickerhamii | None reported | IV amphotericin B + oral doxycycline (induction) → oral voriconazole (ceased at 2 weeks for intolerance) | All lesions resolved | [41] |
| 67 | 2023 | 78 | woman | Australia | Inflammatory arthritis on prednisone + infliximab; prior disseminated Nocardia infection | Immunocompromised (biologic/corticosteroid therapy) | Isolated right forearm plaque (6 months), no trauma | P. wickerhamii | None reported (past Nocardia infection, not concurrent) | Wide surgical excision + split skin graft; oral posaconazole ceased after 1 week (intolerance/LFT derangement); amphotericin B not used | Surgical excision completed; further antifungal not tolerated | [41] |
| 68 | 2024 | 57 | woman | China | Agricultural hand injury (1 year prior); mild lymphopenia, decreased CD3/CD4 T-cells, low albumin (HIV negative) | Mildly immunocompromised | Swelling of dorsum of left hand (>1 year), previously excised and misdiagnosed as cryptococcosis, recurred with ulceration and purulent discharge (1 month) | P. wickerhamii | None reported | Oral itraconazole 200 mg bid + 4 sessions ALA-PDT (weekly) + wound care, total 9-week course | Complete wound healing by 4 months, no recurrence at 2 years | [42] |
| 69 | 2024 | 75 | man | China | COPD, hypertension, chronic active hepatitis B; finger trauma | Immunocompromised (COPD, chronic hepatitis B) | Edema, skin ulcers and pus from right hand to forearm after finger trauma (>1 month), unresponsive to anti-infective therapy and debridement | Not confirmed by culture (histology only) | None reported | IV itraconazole 200 mg qd × 2 weeks (failed) → IV fluconazole 200 mg qd × 3 months | Significant improvement at 3 months | [43] |
| 70 | 2024 | 81 | woman | Brazil | Type 2 diabetes mellitus (metformin); frequent garden plant contact, no trauma | Immunocompromised (diabetes) | Painful erythematous-to-violaceous patches/plaques with erosion and crusting on upper chest/shoulders (1 year) | Not identified to species (culture negative, bacterial overgrowth) | None reported | Itraconazole 200 mg/day | Satisfactory improvement at 6 months | [44] |
| 71 | 2024 | 71 | man | USA | Coronary artery disease (stented), CHF with ICD; hand laceration; later found somatic ASXL1/TET2 variants leading to myelodysplastic syndrome | Immunocompetent at onset (developed MDS during treatment course) | Painful nodules on dorsal right 2nd/3rd digits with sporotrichoid spread up the arm after laceration | P. wickerhamii | None reported | Itraconazole (failed) → voriconazole (relapsed) → posaconazole (partial) → oral lipid nanocrystal amphotericin B (MAT2203), monotherapy × 1 year total | Marked clinical/radiographic improvement (PET/CT resolution); unrelated MDS diagnosed during follow-up | [45] |

**References**

1. Woolrich A, Koestenblatt E, Don P, Szaniawski W. Cutaneous protothecosis and AIDS. J Am Acad Dermatol 1994;31:920-4.

2. Tang WYM, Lo KK, Lam WY, Fung KSC, Koehler A, Cheng AFB. Cutaneous protothecosis: report of a case in Hong Kong. Br J Dermatol 1995;133:479-82.

3. Monopoli A, Accetturi MP, Lombardo GA. Cutaneous protothecosis. Int J Dermatol 1995;34:766-7.

4. Carey WP, Kaykova Y, Bandres JC, Sidhu GS, Bräu N. Cutaneous protothecosis in a patient with AIDS and a severe functional neutrophil defect: successful therapy with amphotericin B. Clin Infect Dis 1997;25:1265-6.

5. Follador I, Bittencourt A, Duran F, Araújo MG. Cutaneous protothecosis: report of the second Brazilian case. Rev Inst Med Trop Sao Paulo 2001;43(5):287-90.

6. Chao SC, Hsu MML, Lee JYY. Cutaneous protothecosis: report of five cases. Br J Dermatol 2002;146:688-93.

7. Leimann BC, Monteiro PC, Lazéra M, Candanoza ER, Wanke B. Protothecosis. Med Mycol 2004;42(2):95-106.

8. Zaitz C, Godoy AM, Colucci FM, de Sousa VM, Ruiz LRB, Masada AS, Nobre MV, Muller H, Muramatu LH, Arrigada GLH, Heins-Vaccari EM, Martins JEC. Cutaneous protothecosis: report of a third Brazilian case. Int J Dermatol 2006;45:124-6.

9. Humphrey S, Martinka M, Lui H. Cutaneous protothecosis following a tape-stripping injury. J Cutan Med Surg 2009;13(5):273-5.

10. Yamada N, Yoshida Y, Ohsawa T, Takahara M, Morino S, Yamamoto O. A case of cutaneous protothecosis successfully treated with local thermal therapy as an adjunct to itraconazole therapy in an immunocompromised host. Med Mycol 2010;48:643-6.

11. Chen Y, Gao A, Ke Y, Zhou X, Lin L, Lu S, Liu Y. Successful treatment of cutaneous protothecosis due to Prototheca wickerhamii with terbinafine. Clin Cosmet Investig Dermatol 2024;17:913-9.

12. Zhang QQ, Li L, Zhu LP, Zhao Y, Wang YR, Zhu JH, Zhu M. Cutaneous protothecosis in patient with diabetes mellitus and review of published case reports. Mycopathologia 2012;173(2-3):163-71.

13. Srisuttiyakorn C, Sindhuphak W. Cutaneous protothecosis: a case report from Thailand. Int J Dermatol 2012;51(11):1340-2.

14. Lu S, Xi L, Qin W, Luo Y, Lu C, Li X. Cutaneous protothecosis: two new cases in China and literature review. Int J Dermatol 2012;51(3):328-31.

15. Gomes-da-Silva PC, Costa-e-Silva SB, Lima RB, D'Acri AM, Lupi O, Martins CJ. Cutaneous protothecosis - case report. An Bras Dermatol 2013;88(6 Suppl 1):183-5.

16. Kwong JC, Ward PB, Johnson PD. Cutaneous protothecosis in a patient with hypogammaglobulinemia. Med Mycol Case Rep 2013;2:132-3.

17. Seok JY, Lee Y, Lee H, Yi SY, Oh HE, Song JS. Human cutaneous protothecosis: report of a case and literature review. Korean J Pathol 2013;47(6):575-8.

18. Fong K, Tee SI, Ho MSL, Pan JY. Cutaneous protothecosis in a patient with previously undiagnosed HIV infection. Australas J Dermatol 2015;56(2):e71-3.

19. Yun CH, Jeong JH, Ryu HR, Kim JH, Baek JO, Lee JR, Seo YH, Roh JY. Cutaneous protothecosis responds rapidly to voriconazole. Int J Dermatol 2015. doi:10.1111/ijd.13160.

20. Woo JY, Suhng EA, Byun JY, Choi HY, Sung SH, Choi YW. A case of cutaneous protothecosis in an immunocompetent patient. Ann Dermatol 2016;28(2):273-5.

21. Nguyen QGL, Rosen T. Cutaneous protothecosis in a patient with chronic lymphocytic leukemia: a case report and literature review. J Fungi 2015;1(1):4-12.

22. Zhang Q, Li L, Yuli K, Zhao Y, Zhu J, Zhu M. A case of cutaneous protothecosis mimics eczema. Mycopathologia 2015;179(1-2):163-6.

23. McMullan B, Pollett S, Biswas C, Packham D. Successful treatment of cutaneous protothecosis with liposomal amphotericin and oral itraconazole. Med Mycol Case Rep 2016;12:21-3.

24. Tan M, Pan JY, Chia HY, Oon HH. Cutaneous protothecosis on the bilateral wrists of a food handler. Clin Exp Dermatol 2017;42:72-4.

25. Inoue M, Miyashita A, Noguchi H, Hirose N, Nishimura K, Masuda M, Ihn H. Case report of cutaneous protothecosis caused by Prototheca wickerhamii designated as genotype 2 and current status of human protothecosis in Japan. J Dermatol 2017. doi:10.1111/1346-8138.14010.

26. Tseng HC, Chen CB, Ho JC, Cheng YW, Huang HW, Sun PL, Lee CH. Clinicopathological features and course of cutaneous protothecosis. J Eur Acad Dermatol Venereol 2018;32:1575-83. (20 patients)

27. Jenkinson H, Thelin L, McAndrew R, Jones KM, Talbott LB, Diven D. Cutaneous protothecosis in a patient on ustekinumab for psoriasis. Int J Dermatol 2018;57(11):1394-6.

28. Wang F, Feng P, Lin Y, Chen X, Xu D, Wang Z, Han J. Human cutaneous protothecosis: a case report and review of cases from Mainland China, Hong Kong, and Taiwan. Mycopathologia 2018;183(5):821-8.

29. Liu Z-H. If it's red and scaly look for fungus: remember cutaneous protothecosis. J Eur Acad Dermatol Venereol 2018. doi:10.1111/jdv.14875.

30. El Hoshy K, Abdel-Halim MRE, El-Nabarawy E, Shalaby S. Cutaneous protothecosis as an unusual complication following dermal filler injection: a case report. J Clin Aesthet Dermatol 2019;12(12):13-6.

31. Zhao F, Chen M, Fu Y. Multiple cutaneous infections caused by Prototheca wickerhamii. J Clin Lab Anal 2020;34(11):e23492.

32. Koutsis JJ, Hong E. Protothecosis: a rare cutaneous infection in an army officer returned from deployment to Iraq. Australas J Dermatol 2020.

33. Rajan A, Pai VV, Shukla P. Cutaneous protothecosis in an immunocompetent host. Indian J Dermatol Venereol Leprol 2020;86:414-7.

34. Godofredo VR, Enokihara MMS, Tomimori J, Ogawa MM. Cutaneous protothecosis in kidney transplant recipient. An Bras Dermatol 2020;95(2):210-3.

35. Lu Y, Zhang X, Ni F, Xia W. Cutaneous protothecosis with meningitis due to Prototheca wickerhamii in an immunocompetent teenager: case report and literature review. Infect Drug Resist 2021;14:2787-94.

36. Mithari HS, Gole PV, Darkase B, Kalantri MS, Khopkar U. Cutaneous protothecosis. Indian J Dermatol 2022;67(4):464-5.

37. Melo RR, Rodrigues FT, Baka JLCS, Almeida MD, Alves MFGS, Gripp AC. An exuberant case of protothecosis: an emergent disease in tropical dermatology. Dermatol Online J 2022;28(1):11.

38. Di Prinzio A, Ruf M, Torre AC, Duran Daza SV, Volonteri VI, Flores V, Mazzuoccuolo LD. Cutaneous protothecosis in a solid organ transplanted patient. An Bras Dermatol 2023;98(5):723-4.

39. Santos DWCL, Diniz YCM, da Silva RR, Marques SG, Bomfim MRQ, de Azevedo CMPS. Human protothecosis: a case report in northeastern Brazil. Med Mycol Case Rep 2023;40:1-4.

40. Rodrigues FT, Santos PFAM, Soares JVS, Alves MFGS, Azulay-Abulafia L. Two cases of protothecosis in Brazil: when it is not a bacterial, fungal or viral dermatosis. J Dtsch Dermatol Ges 2023;21(9):1022-4. (2 patients)

41. Orschulok T, Whitfield J, Badrick T, Sivabalan P, Sowden D, Lehane F. Cutaneous protothecosis: contrasting case presentations. Pathology 2023;55(7):1032-5. (2 patients)

42. Cai W, Huang J, Zhang Q, Li J, Lin L, Zhang J, Xi L, Lu S. Combinatory effect of ALA-PDT and itraconazole in the treatment of cutaneous protothecosis. Photodiagnosis Photodyn Ther 2024;49:104332.

43. Xu C, Pan C, Xu C, Zheng W, Yin Q, Pan H, Chen Y. Successful treatment of cutaneous protothecosis with fluconazole: a case report and epidemiology study of Prototheca infection in China. J Cosmet Dermatol 2024;23(6):2190-8.

44. Aguinaga F, Carranza K, Azulay V, Zarco OM, Quintella DC, Cuzzi T. Cutaneous protothecosis: a new case and review of Brazilian patients. Dermatol Online J 2024;30(5):7.

45. Pechacek J, Schmitt MM, Ferrè EMN, Webb T, Goldberg J, Pathan S, Banerjee C, Barber P, DiMaggio T, Quinn A, Matkovits T, Castelo-Soccio L, Nussenblatt V, Lionakis MS. Successful treatment of refractory cutaneous protothecosis with MAT2203, an oral lipid nanocrystal formulation of amphotericin B. Open Forum Infect Dis 2024.
